# Lactotransferrin in Asian Elephant (*Elephas maximus*) Seminal Plasma Correlates with Semen Quality

**DOI:** 10.1371/journal.pone.0071033

**Published:** 2013-08-16

**Authors:** Wendy K. Kiso, Vimal Selvaraj, Jennifer Nagashima, Atsushi Asano, Janine L. Brown, Dennis L. Schmitt, John Leszyk, Alexander J. Travis, Budhan S. Pukazhenthi

**Affiliations:** 1 Center for Species Survival, Smithsonian Conservation Biology Institute, National Zoological Park, Washington, District of Columbia, United States of America; 2 Department of Environmental Science and Policy, George Mason University, Fairfax, Virginia, United States of America; 3 Department of Animal Science, Cornell University, Ithaca, New York, United States of America; 4 The Baker Institute for Animal Health, College of Veterinary Medicine, Cornell University, Ithaca, New York, United States of America; 5 Center for Species Survival, Smithsonian Conservation Biology Institute, National Zoological Park, Front Royal, Virginia, United States of America; 6 The William H. Darr School of Agriculture, Missouri State University, Springfield, Missouri, United States of America; 7 The Ringling Bros. Center for Elephant Conservation, Polk City, Florida, United States of America; 8 University of Massachusetts Medical School Proteomics and Mass Spectrometry Facility, University of Massachusetts Medical School, Shrewsbury, Massachusetts, United States of America; CNRS, France

## Abstract

Asian elephants (*Elephas maximus*) have highly variable ejaculate quality within individuals, greatly reducing the efficacy of artificial insemination and making it difficult to devise a sperm cryopreservation protocol for this endangered species. Because seminal plasma influences sperm function and physiology, including sperm motility, the objectives of this study were to characterize the chemistry and protein profiles of Asian elephant seminal plasma and to determine the relationships between seminal plasma components and semen quality. Ejaculates exhibiting good sperm motility (≥65%) expressed higher percentages of spermatozoa with normal morphology (80.3±13.0 vs. 44.9±30.8%) and positive Spermac staining (51.9±14.5 vs. 7.5±14.4%), in addition to higher total volume (135.1±89.6 vs. 88.8±73.1 ml) and lower sperm concentration (473.0±511.2 vs. 1313.8±764.7×10^6^ cells ml^−1^) compared to ejaculates exhibiting poor sperm motility (≤10%; *P*<0.05). Comparison of seminal plasma from ejaculates with good versus poor sperm motility revealed significant differences in concentrations of creatine phosphokinase, alanine aminotransferase, phosphorus, sodium, chloride, magnesium, and glucose. These observations suggest seminal plasma influences semen quality in elephants. One- and two-dimensional (2D) gel electrophoresis revealed largely similar compositional profiles of seminal plasma proteins between good and poor motility ejaculates. However, a protein of ∼80 kDa was abundant in 85% of ejaculates with good motility, and was absent in 90% of poor motility ejaculates (*P*<0.05). We used mass spectrometry to identify this protein as lactotransferrin, and immunoblot analysis to confirm this identification. Together, these findings lay a functional foundation for understanding the contributions of seminal plasma in the regulation of Asian elephant sperm motility, and for improving semen collection and storage in this endangered species.

## Introduction

The captive North American Asian elephant population is not self-sustaining; breeding pairs are not producing adequate numbers of offspring to maintain a stable population demographic. Thus, a major goal of captive elephant conservation efforts is to increase offspring production by either natural or assisted reproduction [1]. Although natural breeding is encouraged, it is often not possible due to various factors, including geographic distance or behavioral incompatibility. Hence, optimizing semen collection techniques and establishing a genome resource bank (GRB), paralleled with the use of artificial insemination (AI), would have tremendous value in the preservation and genetic management of the endangered Asian elephant [2].

Obtaining consistently high quality ejaculates from Asian elephants has been a challenge, with ejaculates demonstrating great variation and a high proportion (> 85%) exhibiting reduced semen quality [3]. This high incidence of variability in ejaculate quality is not only observed among bulls (including bulls of known fertility), but also among ejaculates from the same bull, even if collected on the same day. This lack of consistency and availability of good quality ejaculates has reduced the utility of AI, and also has been a major impediment towards optimizing sperm cryopreservation and establishing a GRB for Asian elephants [4]. Therefore, there is an urgent need to better understand the physiological basis for good versus poor ejaculate quality in elephants.

Semen from elephants has been collected using a variety of methods including electroejaculation [5], manual stimulation [6], artificial vagina [7], rectal massage without sedation [8], [9], [10], and rectal massage with standing sedation [11]. The rectal massage method of semen collection [8] has been adopted at many bull-holding facilities due to its safety, practicality, and ability to collect bull elephants without sedation. Although good quality ejaculates can be collected using this method, urine contamination has been a major factor compromising overall semen quality. In addition, it is conceivable that this semen collection method does not trigger appropriate contributions from all the accessory sex glands, which in the elephant include the ampullae, seminal vesicles, prostate glands, and bulbourethral glands [12]. Because seminal plasma has been found to influence many aspects of sperm function and physiology, including sperm motility and the acquisition of fertilization competence [13], [14], [15], [16], we hypothesized that variable contributions from one or more accessory sex glands during the collection process might be influencing semen quality.

To test this hypothesis, we utilized biochemical analysis and mass spectrometry-based proteomics to: i) evaluate the chemical and protein profiles of Asian elephant seminal plasma; ii) compare seminal plasma chemistry and protein profiles of good (≥65% total sperm motility) versus poor (≤10% total sperm motility) quality ejaculates; and iii) identify seminal plasma proteins that correlate with good sperm motility in an ejaculate. Increasing our understanding of male reproduction in Asian elephants could potentially lead to improvements in semen collection methods, and extender/cryopreservation media to optimize use of assisted reproduction in, and conservation of, the endangered Asian elephant.

## Materials and Methods

### Animals

Seminal plasma was collected from Asian elephant bulls (n = 21; 8 to 45 years) housed at 10 institutions throughout North America. Sixteen of the 21 bulls had previously sired calves and were therefore known to be fertile by natural mating. The bulls were managed under a protected contact management program, housed in individual enclosures with visual, olfactory, and/or controlled access to females, and given free access to water and regular access to feed. All animal research protocols were approved by the Smithsonian Conservation Biology Institute's Institutional Animal Care and Use Committee.

### Semen Collection and Processing

Semen was collected using the rectal massage technique as previously described [8]. Each ejaculate (n = 21 bulls; 205 ejaculates; 1–22 ejaculate(s) per bull) was immediately evaluated for volume (ml), color, percentages of total motile spermatozoa (% tMOT) and forward progressive motility (% pMOT), sperm concentration (×10^6^ cells ml^−1^), sperm morphology, osmolality, and pH. An aliquot (8 µl) was assessed subjectively for % tMOT and % pMOT using a phase contrast microscope (200X). Sperm concentration was determined using a portable spectrophotometer (DVM Rapid Test^TM^, Value Diagnostics) calibrated for measuring concentration of Asian elephant spermatozoa. Osmolality (mOsm) was determined using a vapor pressure osmometer (VAPRO, Wescor Inc.) and pH was determined using a hand held pH meter (Twin pH, Horiba Ltd.).

**Figure 1:**
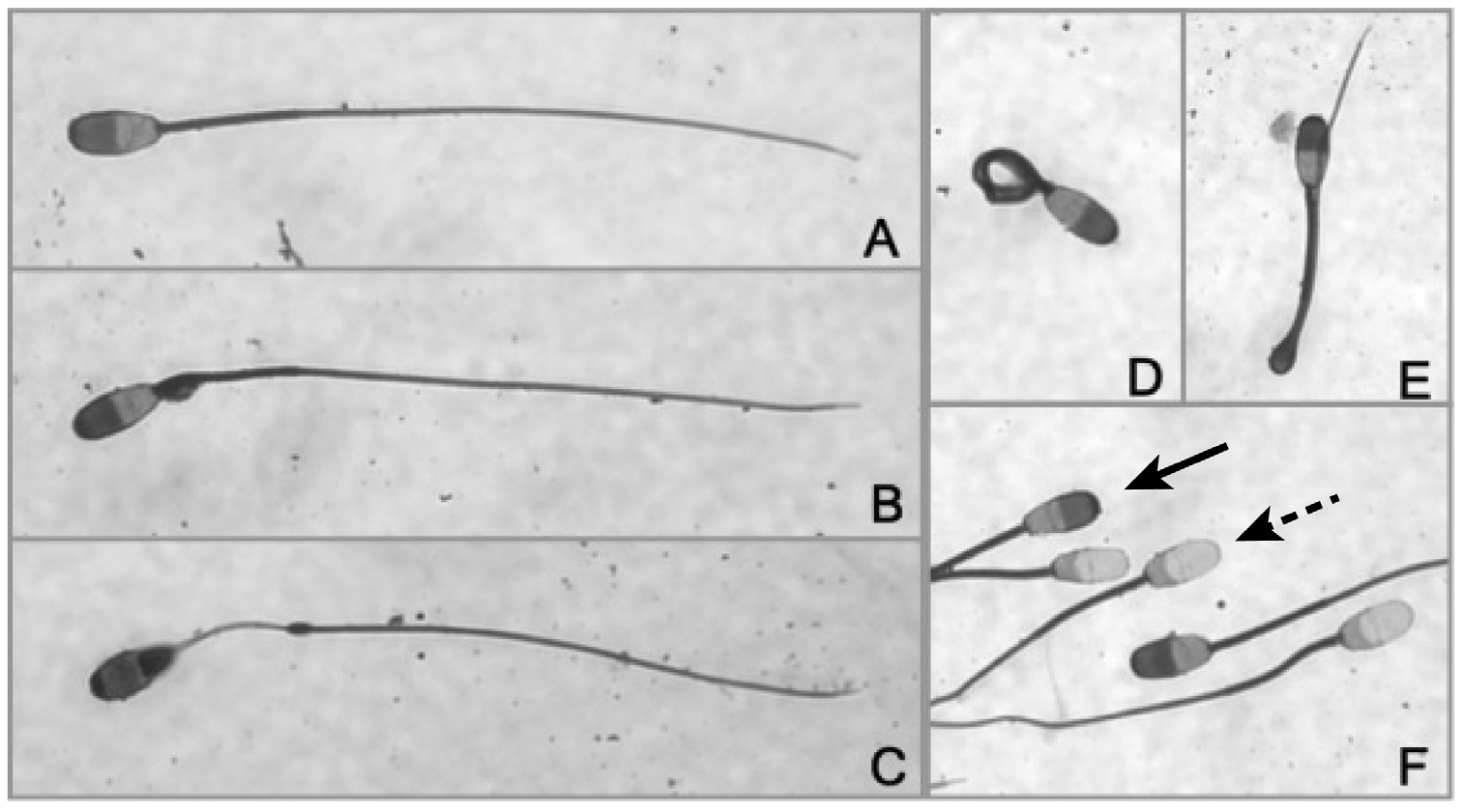
Asian elephant sperm morphology. A) Normal; B) Proximal cytoplasmic droplet; C) Abnormal mid-piece; D) Tightly coiled tail; E) Bent tail with cytoplasmic droplet; F) Spermac staining (solid arrow: Spermac positive; dotted arrow: Spermac negative). Magnification 1000X.

Sperm morphology was evaluated using Spermac stain (Conception Technologies) as previously described [3]. For morphological assessment, a minimum of 200 spermatozoa were assessed individually using bright-field microscopy under oil immersion (1000X). Spermatozoa exhibiting normal morphology were categorized as ‘normal’ (Figure 1A), and spermatozoa exhibiting morphological abnormalities in the head (i.e. microcephalic, macrocephalic, bicephalic, misshaped, detached), mid-piece (i.e. bent necks, abnormal, bent, absent, proximal or distal cytoplasmic droplet), or flagellum (i.e. double, coiled, bent) were categorized as ‘abnormal’ (e.g. Figure 1B–E). For evaluation of the rostral sperm head, an additional 200 spermatozoa (minimum) were evaluated under oil immersion (1000X) and individually categorized as being Spermac positive or negative (Figure 1F). Spermatozoa that exhibited uniform staining at the rostral head were categorized as ‘Spermac positive’. Spermatozoa that exhibited non-uniform staining, lack of staining, or appeared vesiculated at the rostral head were categorized as ‘Spermac negative’. The numbers of spermatozoa with normal morphology and positive Spermac staining in the region of the acrosome were each converted into a percentage.

Ejaculates with overt visual or olfactory signs of urine contamination were not included in this study (113 ejaculates; 18 bulls; 1–13 ejaculate(s) per bull). For isolation of seminal plasma, each ejaculate (92 ejaculates; 11 bulls; 1–17 ejaculate(s) per bull) was initially centrifuged (200×*g* for 5 min) to pellet spermatozoa and cellular debris. The seminal plasma supernatant was removed and centrifuged again (500×*g* for 20 min), with the top 2/3 removed by aspiration, mixed, divided into aliquots, frozen and stored (−70°C) until analysis. After thawing, all aliquots were spun additionally at 10,000×*g* for 5 min at 4°C and the supernatants collected to ensure that all analyzed samples were devoid of spermatozoa.

### Seminal Plasma Chemistry Analyses

Seminal plasma electrolytes (Na^+^: sodium; P^3−^: phosphorus; K^+^: potassium; Ca^2+^: calcium; Cl^−^: chloride; HCO_3_^−^: bicarbonate), enzymes (LDH: lactate dehydrogenase; CPK: creatine phosphokinase; AST: aspartate aminotransferase; ALT: alanine aminotransferase; AP: alkaline phosphatase), proteins (TP: total protein; ALB: albumin), sugars (GLU: glucose), cholesterol (CHO), creatinine (CRT), and urine urea nitrogen (UUN) were determined using a serum chemistry autoanalyzer (Roche Cobas Mira Chemistry Analyzer). Although ejaculates with definitive signs of urine contamination were excluded from this study, CRT and UUN levels were also measured to identify low levels of urine contamination. Magnesium (Mg^2+^) concentrations were measured by a colorimetric method using a Hitachi Cobas C501 chemistry analyzer (performed at the Kansas State Veterinary Diagnostic Laboratory).

### Seminal Plasma Protein Analyses

#### Protein concentration and precipitation

Total protein (TP) concentration of seminal plasma was determined using the Bicinchoninic Acid (BCA) assay (Micro BCA^TM^ Protein Assay Kit, Pierce Biotechnology) according to the manufacturer's instructions. Prior to separation by SDS-PAGE, proteins were precipitated to concentrate the samples. For protein precipitation, cold trichloroacetic acid (TCA; 4% final concentration) was added to seminal plasma samples, centrifuged (10,000×*g* for 20 min; 4°C), the supernatant removed and the protein pellet was washed with cold acetone and centrifuged (10,000×*g* for 10 min; 3 times) to remove excess TCA.

#### SDS polyacrylamide gel electrophoresis (SDS-PAGE)

SDS-PAGE, used for separation and determination of molecular weights of seminal plasma proteins, was performed on vertical 0.75 mm 10% polyacrylamide gels. Samples (50 μg of protein) were boiled for 5 min in sample buffer [17] before separation by SDS-PAGE. Electrophoresis was run at 5 mA per gel (∼12 h), after which gels were fixed in 25% (v/v) isopropanol and 10% (v/v) acetic acid and the proteins were visualized by incubation in Coomassie blue [0.06% (w/v) Coomassie brilliant blue G250, 10% (v/v) acetic acid]. Gels were rinsed and stored in 10% (v/v) acetic acid for 5–15 min until evaluation. For quantification of the protein band corresponding to lactotransferrin (80 kDa band), gels were scanned and analyzed using ImageJ (ImageJ 1.47t, National Institutes of Health, http://imagej.nih.gov/ij) among good vs. poor motility samples. Band intensities were normalized relative to the total intensity of all protein loaded per lane, and were analyzed as described below (Statistical Analysis section).

#### Two dimensional gel electrophoresis (2D gels)

High resolution 2D gels were used to separate elephant seminal plasma proteins. Briefly, following precipitation, proteins were resuspended in 2D electrophoresis rehydration solution [8 M urea, 2 M thiourea, 2% (w/v) ASB-C8φ, 0.5% (v/v) IPG buffer, 18 mM dithiothreitol (DTT), 0.002% (w/v) bromophenol blue]. Isoelectric focusing (IEF) was carried out using immobilized pH gradient (IPG) gel strips (Immobiline^TM^ DryStrip; pH 3–10 NL, 24 cm; Amersham Biosciences) on a horizontal Ettan IPGphor Isoelectric Focusing System (Amersham Biosciences). Proteins entered the first dimension during rehydration under a low voltage (30V) for 12 h to minimize aggregation and facilitate entry of higher molecular weight proteins. After rehydration, voltage was applied at 500V for 1 h, 1000V for 1 h, and 8000V for 8.2 h, for a total of 22.2 h.

Following overnight IEF, IPG gel strips were incubated in equilibration buffer [6 M urea, 75 mM Tris-HCl, 30% (v/v) glycerol, 2% (w/v) SDS, 0.002% (w/v) bromophenol blue with 1% (w/v) DTT] for 15 min, followed by the same buffer without DTT and supplemented with 2.5% (w/v) iodoacetamide for an additional 15 min. After equilibration, IPG gel strips and molecular weight protein markers (Bio-Rad) were laid onto 25.5×19.6 cm, 1.5 mm thickness, 10–12% second-dimension (2D) polyacrylamide gradient gels. A 1% (w/v) agarose solution with 0.002% (w/v) bromophenol blue was laid on top of IPG gel strips and 2D gels to ensure IPG gel strips remained in stable contact with the gels. The second dimension gels were then subjected to electrophoresis (8 mA per gel for 20–22 h or 10 mA per gel for 10–11 h) on an Ettan DALT*twelve* Vertical System (Amersham Biosciences).

After electrophoresis, gels were fixed and stained for protein visualization using either Coomassie blue or silver staining. Coomassie blue was performed as described above for protein visualization on SDS-PAGE gels. Silver staining was performed with slight modifications as described previously by Morrissey [18]. Briefly, the gels were placed on an orbital shaker and incubated in fixative [50% (v/v) methanol and 10% (v/v) acetic acid] for 20 min and refreshed with additional fixative for another 20 min. The gels were rinsed in 20% (v/v) ethanol for 10 min, washed in Milli-Q water for an additional 10 min, and placed in reducing solution [0.02% (v/v) sodium thiosulfate] for 1 min. Gels were rinsed twice with Milli-Q water followed by incubation in 0.2% (w/v) silver nitrate solution for 30 min in the dark. After incubation in silver nitrate, gels were rinsed in Milli-Q water. Developing solution [3% (w/v) sodium carbonate, 37% formaldehyde, and 0.001% sodium thiosulfate] was added to gels until proteins were visualized with desired intensity (∼30 seconds) after which gels were quickly rinsed in 1% (v/v) acetic acid to stop exposure. Selected protein spots were excised and stored at −70°C until mass spectrometry analysis.

#### Protein identification by mass spectrometry

Excised protein spots were digested “in gel” with trypsin. Since the elephant genome was not known at the time of analysis we derivitized the tryptic peptides with 4-sulphophenyl isothiocynate (SPITC) to facilitate *de novo* sequencing of Post-Source Decay (PSD) tandem mass spectra. Briefly dried protein digests were dissolved in 8.5 µl of SPITC solution (10 mg/ml in 20 mM NaHCO_3_, pH 9.5). The sample was incubated for 30 min at 55°C on a heating block. The reaction was stopped by the addition of 4.5 µl of 5% trifluoroacetic acid (TFA). Samples were further concentrated and desalted using micro C_18_ ZipTips (Millipore, Inc.) prior to MALDI TOF (Matrix Assisted Laser Desorption/Ionization Time-of-Flight) analysis mass spectrometry (Shimadzu Biotech Axima TOF^2^). PSD spectra were manually interpreted with the aid of Mascot Distiller v 2.1 (Matrix Sciences, Ltd.). *De novo* sequences were searched against the NCBI nr protein database using the BLAST program. More recently, the genome of the African elephant (*Loxodonta africana*) has been determined by the Broad Institute (http://www.broadinstitute.org). A Blast search of the 4 *de novo* determined sequences was performed against the predicted protein sequence database of *Loxodonta africana*. Mass spectrometry identification was done at the Proteomic Mass Spectrometry Laboratory at the University of Massachusetts Medical School.

#### Immunoblotting for detection of lactotransferrin

For detection of lactotransferrin in elephant seminal plasma, seminal plasma proteins were separated by SDS-PAGE followed by protein immunoblotting as previously described by Travis et al. [19], with slight modifications. After SDS-PAGE, proteins were transferred onto Immobilon-P membranes (Millipore, Inc.). Membranes were blocked for at least 30 min in 5% (w/v) nonfat skim milk in a Tris-buffered saline solution containing 0.1% Tween 20 (TTBS) and incubated overnight with primary antibody (1∶50 dilution; anti-transferrin goat polyclonal antibody; product no. sc-22595; Santa Cruz Biotechnology). A polyclonal antibody with cross-reactivity to transferrin from different species was utilized because of high homologies among species and the lack of an elephant-specific lactotransferrin antibody. Blots were then washed in TTBS and incubated in peroxidase-conjugated secondary antibody (polyclonal rabbit anti-goat immunoglobulin/HRP, DakoCytomation) for 1 h (1∶3000 dilution). Blots were rinsed in TTBS and developed using chemiluminescence.

### Statistical Analysis

Ejaculate traits and seminal plasma constituents are presented as mean ± SD. All data were analyzed using SAS 9.1 (SAS Institute Inc.). Significant differences in ejaculate traits and seminal plasma components between ejaculates exhibiting good (≥65% tMOT) vs. poor (≤10% tMOT) sperm motility were analyzed using Student's t-test mean comparison procedures. Pearson product-moment correlation coefficients were calculated to determine associations between seminal plasma components and semen parameters. Student's t-test was utilized to determine differences in 80 kDa band intensities among good vs. poor sperm motility samples. Differences were considered significant at *P<*0.05.

## Results

### Semen Characteristics

Although all bulls produced spermic ejaculates (n = 21 bulls; 205 ejaculates), 55% (113/205) of ejaculates were not included in the study due to overt urine contamination. The average percent values (mean ± SD) of traits for the remaining ejaculates (n = 11 bulls; 92 ejaculates) without obvious urine contamination are summarized in Table 1 (Category: All Ejaculates). The characteristics of the 92 ejaculates evaluated demonstrated a wide range in values for all traits, including volume, sperm concentration, sperm motility (% tMOT, % pMOT), normal sperm morphology, Spermac staining, osmolality, and pH (Table 1). When taken as a whole, the mean % tMOT (31.3±36.3%), % pMOT (28.0±35.3%), and positive Spermac staining (24.0±26.1%) appeared to be low. In Table 1, all five bulls that contributed to the good motility sub-group also contributed to the poor motility sub-groups showing the variation in quality within each individual. All traits except osmolality were significantly different (*P*<0.05) between good (≥65% tMOT) and poor (≤10% tMOT) motility samples (Table 1). Ejaculate volume was higher (*P*<0.05) in good motility samples, and sperm concentration was higher (*P*<0.05) in poor motility samples (Table 1). Furthermore, good motility samples exhibited higher (*P*<0.05) values in normal morphology and positive Spermac staining over the acrosomes compared to their poor motility counterparts (Table 1).

**Table 1:** Characteristics of Asian elephant ejaculates and comparison of semen traits between good and poor motility ejaculates.

	All Ejaculates			Good Motility			Poor Motility		
	(92 ejaculates; 11 bulls)			(28 ejaculates; 5 bulls)			(52 ejaculates; 10 bulls)		
Semen Trait	Mean	± SD	(Range)	Mean	± SD	(Range)	Mean	± SD	(Range)
Total volume (ml)	98.5	±79.6	(3–346)	135.1	±89.6 ^a^	(20–346)	88.8	±73.1 ^b^	(7.5–310)
Sperm concentration (×10^6^ cells/ml)	1008.2	±800.8	(9–2665)	473.0	±511.2 ^a^	(10–2000)	1313.8	±764.7 ^b^	(2–2665)
% Total motility (% tMOT)	31.3	±36.3	(0–95)	81.3	±9.1 ^a^	(65–95)	1.9	±3.6 ^b^	(0–10)
% Progressive motility (% pMOT)	28.0	±35.3	(0–95)	75.2	±18.8 ^a^	(10–95)	1.2	±3.1 ^b^	(0–10)
Normal sperm (%)	59.5	±28.6	(0–95)	80.3	±13.0 ^a^	(47–95)	44.9	±30.8 ^b^	(2–88)
Spermac positive (%)	24.0	±26.1	(0–87)	51.9	±14.5 ^a^	(31–70)	7.5	±14.4 ^b^	(0–79)
Osmolality (mOsm)	271.7	±62.2	(112–537)	265.4	±12.1	(242–291)	282.8	±103.5	(112–537)
pH	6.8	±0.8	(4.9–8.3)	7.07	±0.5 ^a^	(6.00–8.29)	6.50	±0.9 ^b^	(4.89–7.81)

Good Motility ejaculates: ≥65% tMOT; Poor Motility ejaculates: ≤10% tMOT.

a,b Within a row, means with different superscripts between good versus poor motility ejaculates differ (*P*<0.05).

Ejaculates with overt visual or olfactory signs of urine contamination were not included.

### Seminal Plasma Chemistry Analyses

A total of 61 seminal plasma samples from nine bulls were analyzed. All five bulls that contributed to the good motility sub-group also contributed to the poor motility sub-groups. Mean seminal plasma electrolytes (Ca^2+^, P^3−^, Na^+^, K^+^, Cl^−^, Mg^2+^, and HCO_3_^−^), enzymes (LDH, CPK, AST, ALT, and AP), cholesterol (CHO), sugar (GLU), protein (TP and ALB), and urine markers (CRT and UUN) are summarized in Table 2. Comparison between good and poor motility samples revealed significant differences (*P<*0.05) in CPK, ALT, P^3−^, Na^+^, Cl^−^, Mg^2+^, and GLU (Table 2). All other seminal plasma components were similar between good and poor motility ejaculates, including values for CRT and UUN.

**Table 2:** Summary statistics for seminal plasma components^1^.

	All Ejaculates				Good Motility				Poor Motility			
	(9 Bulls; Except: ^‡^6 Bulls, *4 Bulls)				(5 Bulls; Except: *4 Bulls, ^†^3 Bulls)				(9 Bulls; Except: ^∞^5 Bulls,*4 Bulls)			
	N	Mean	± SD	(Range)	N	Mean	± SD	(Range)	N	Mean	± SD	(Range)
**TP**	61	6.87	±4.3	(0–20)	24	4.67	±4.50	(0–20)	36	8.23	±3.7	(10–15)
**ALB**	60	1.52	±1.0	(0–4)	23	1.3	±1.1	(0–3)	36	1.6	±0.9	(0–4.0)
**LDH**	31	24.68	±52.2	(0–190)	10*	6.20	±15.11	(0–49)	21	33.48	±61.10	(0–190)
**CPK**	59	7.27	±11.9	(0–55)	22	11.86	±13.12 ^a^	(0–51)	36	3.97	±9.80 ^b^	(0–55)
**AST**	61	10.98	±12.2	(0–60)	24	9.21	±12.47	(0–59)	36	12.33	±12.23	(0–60)
**ALT**	61	4.95	±4.2	(0–18)	24	3.46	±2.67 ^a^	(0–10)	36	5.92	±4.79 ^b^	(0–18)
**AP**	60	500.78	±615.9	(3–3125)	23	465.65	±701.39	(3–3125)	36	462.08	±435.24	(3–1688)
**Ca^2+^**	61	13.87	±19.9	(1–98)	24	8.81	±17.36	(1–80)	36	17.45	±21.20	(2–98)
**P^3−^**	61	5.82	±5.6	(0–21)	24	2.74	±3.87 ^a^	(0–19)	36	7.44	±5.25 ^b^	(0–18)
**Na^+^**	59	85.25	±36.4	(10–150)	22	109.64	±28.11 ^a^	(21–129)	36	70.47	±33.43 ^b^	(10–150)
**K^+^**	59	22.03	±17.7	(3–94)	22	18.95	±18.84	(3–94)	36	24.18	±17.06	(9–78)
**Cl^−^**	59	89.76	±41.4	(14–250)	22	107.14	±27.36 ^a^	(38–135)	36	80.50	±45.19 ^b^	(14–250)
**Mg^2+^**	50	4.82	±6.2	(0–32)	20	2.22	±2.97 ^a^	(0–13)	29	6.66	±7.20 ^b^	(1–32)
**GLU**	59	3.44	±6.6	(0–37)	22	6.45	±9.58 ^a^	(0–37)	36	1.42	±2.35 ^b^	(0–10)
**CHO**	24^‡^	13.17	±12.3	(0–41)	12*	10.50	±10.48	(0–41)	11^∞^	13.73	±12.31	(3–38)
**HCO_3_^−^**	35	5.80	±5.6	(0–22)	10*	6.60	±3.24	(3–10)	25	5.48	±6.29	(0–22)
**CRT**	61	5.06	±7.6	(0–46)	24	3.93	±10.03	(0–46)	36	5.94	±5.44	(0–28)
**UUN**	16*	96.69	±78.9	(13–288)	6^†^	85.83	±113.50	(13–288)	10*	103.20	±55.71	(24–187)

1 Seminal Plasma Components: Total Protein (TP), mg/ml; Albumin (ALB), mg/ml; Lactate dehydrogenase (LDH), U/L; Creatine phosphokinase (CPK), U/L; Aspartate aminotransferase (AST), U/L; Alanine aminotransferase (ALT), U/L; Alkaline phosphatase (AP), U/L; Calcium (Ca^2+^), mg/dl; Phosphorus (P^3−^), mg/dl; Sodium (Na^+^), mmol/L; Potassium (K^+^), mmol/L; Chloride (Cl^−^), mmol/L; Magnesium (Mg^2+^), mg/dl; Glucose (GLU), mg/dl; Cholesterol (CHO), mg/dl; Bicarbonate (HCO_3_^−^), mmol/L; Creatinine (CRT), mg/dl; Urea nitrogen (UUN), mg/dl.

Good Motility ejaculates: ≥65% tMOT; Poor Motility ejaculates: ≤10% tMOT.

a,b Within a row, means with different superscript between good versus poor motility ejaculates differ (*P*<0.05).

Correlation analysis between semen characteristics and seminal plasma components are summarized in Table 3. Negative correlations were found between volume and TP, AP, and Ca^2+^; whereas volume exhibited a positive correlation with GLU. Although sperm concentration was negatively correlated with Na^+^, it was positively correlated with TP, ALB, AST, ALT, AP, P^3−^, and CHO. Both total and progressive sperm motility were negatively correlated with TP, ALT, Ca^2+^, P^3−^, and Mg^2+^; both were also positively correlated with CPK, Na^+^, Cl^−^, and GLU. Percent normal sperm morphology was negatively associated with Ca^2+^, K^+^, Mg^2+^, and CHO; but exhibited positive correlations with Na^+^ and Cl^−^. Positive Spermac staining was negatively correlated with TP, ALT, Ca^2+^, P^3−^, K^+^, Mg^2+^, CRT, and UUN; and exhibited a positive association with CPK, Na^+^, and GLU. Osmolality exhibited only positive associations with TP, ALT, Ca^2+^, P^3−^, K^+^, Cl^−^, Mg^2+^, and UUN. Semen pH exhibited a positive correlation with HCO_3_^−^; but was negatively correlated with ALT and P^3−^.

**Table 3:** Pearson product-moment coefficients between conventional semen parameters and seminal plasma components^1^.

	Seminal Plasma Component																	
Semen Trait	TP	ALB	LDH	CPK	AST	ALT	AP	Ca^2+^	P^3−^	Na^+^	K^+^	Cl^−^	Mg^2+^	GLU	CHO	HCO_3_^−^	CRT	UUN
Volume	−0.36*	NS	NS	NS	NS	NS	−0.33*	−0.29*	NS	NS	NS	NS	NS	0.38	NS	NS	NS	NS
Sperm Concentration	0.51*	0.40*	NS	NS	0.42*	0.46*	0.48*	NS	0.80*	−0.28*	NS	NS	NS	NS	0.70*	NS	NS	NS
% tMOT	−0.41*	NS	NS	0.34*	NS	−0.29*	NS	−0.47*	−0.44*	0.55*	NS	0.32*	−0.37*	0.38*	NS	NS	NS	NS
% pMOT	−0.43*	NS	NS	0.36*	NS	−0.30*	NS	−0.48*	−0.43*	0.57*	NS	0.32*	−0.35*	0.40*	NS	NS	NS	NS
% Normal Sperm	NS	NS	NS	NS	NS	NS	NS	−0.65*	NS	0.62*	−0.49*	0.76*	−0.52*	NS	−0.86*	NS	NS	NS
% Spermac Positive	−0.39*	NS	NS	0.61*	NS	−0.34*	NS	−0.38*	−0.60*	0.42*	−0.34*	NS	−0.41*	0.36*	NS	NS	−0.35*	−0.79*
Osmolality	0.34*	NS	NS	NS	NS	0.49*	NS	0.69*	0.34*	NS	0.60*	0.71*	0.72*	NS	NS	NS	NS	0.73*
pH	NS	NS	NS	NS	NS	−0.55*	NS	NS	−0.73*	NS	NS	NS	NS	NS	NS	0.35*	NS	NS

1 Seminal Plasma Components: Total Protein (TP), mg/ml; Albumin (ALB), mg/ml; Lactate dehydrogenase (LDH), U/L; Creatine phosphokinase (CPK), U/L; Aspartate aminotransferase (AST), U/L; Alanine aminotransferase (ALT), U/L; Alkaline phosphatase (AP), U/L; Calcium (Ca^2+^), mg/dl; Phosphorus (P^3−^), mg/dl; Sodium (Na^+^), mmol/L; Potassium (K^+^), mmol/L; Chloride (Cl^−^), mmol/L; Magnesium (Mg^2+^), mg/dl; Glucose (GLU), mg/dl; Cholesterol (CHO), mg/dl; Bicarbonate (HCO_3_^−^), mmol/L; Creatinine (CRT), mg/dl; Urea nitrogen (UUN), mg/dl.

* P<0.05; NS: Not significant.

### Seminal Plasma Protein Analyses

#### SDS polyacrylamide gel electrophoresis (SDS-PAGE)

**Figure 2:**
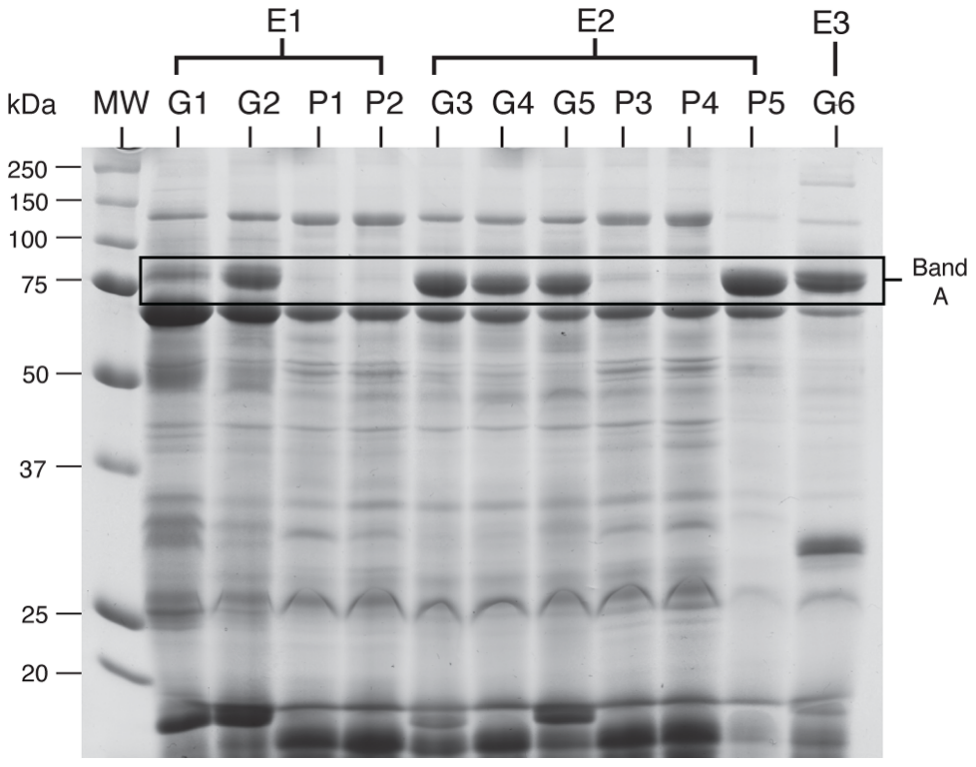
One-dimensional gel electrophoresis of Asian elephant seminal plasma proteins. Each column represents protein profiles from seminal plasma samples obtained from different ejaculates. E1, E2, and E3 denote individual Asian elephant bulls. G1, G2, G3, G4, G5, and G6 denote seminal plasma samples obtained from ejaculates exhibiting good motility (≥65% tMOT). P1, P2, P3, P4, and P5 denote seminal plasma samples obtained from ejaculates exhibiting poor motility (≤10% tMOT). MW: molecular weight marker. Band A was detected in 85% of seminal plasma samples from good motility ejaculates (a representative gel is shown). In this figure, protein bands were visualized with Coomassie staining.

Asian elephant seminal plasma samples (15 ‘good motility’ samples from 5 bulls; 15 ‘poor motility’ samples from 6 bulls) were separated according to molecular weight by SDS-PAGE (Figure 2). A number of proteins were visualized in all samples. However, a prominent band at ∼ 80 kDa (Band A) was observed in 85% of ejaculates exhibiting good motility (Figure 2), but was absent in 90% of poor motility ejaculates (*P*<*0.05*). Quantification of the 80 kDa protein revealed a 2.6-fold higher (*P*<*0.05*) expression in ejaculates exhibiting good motility compared to poor motility counterparts.

#### Two dimensional gel electrophoresis (2D gels)

**Figure 3:**
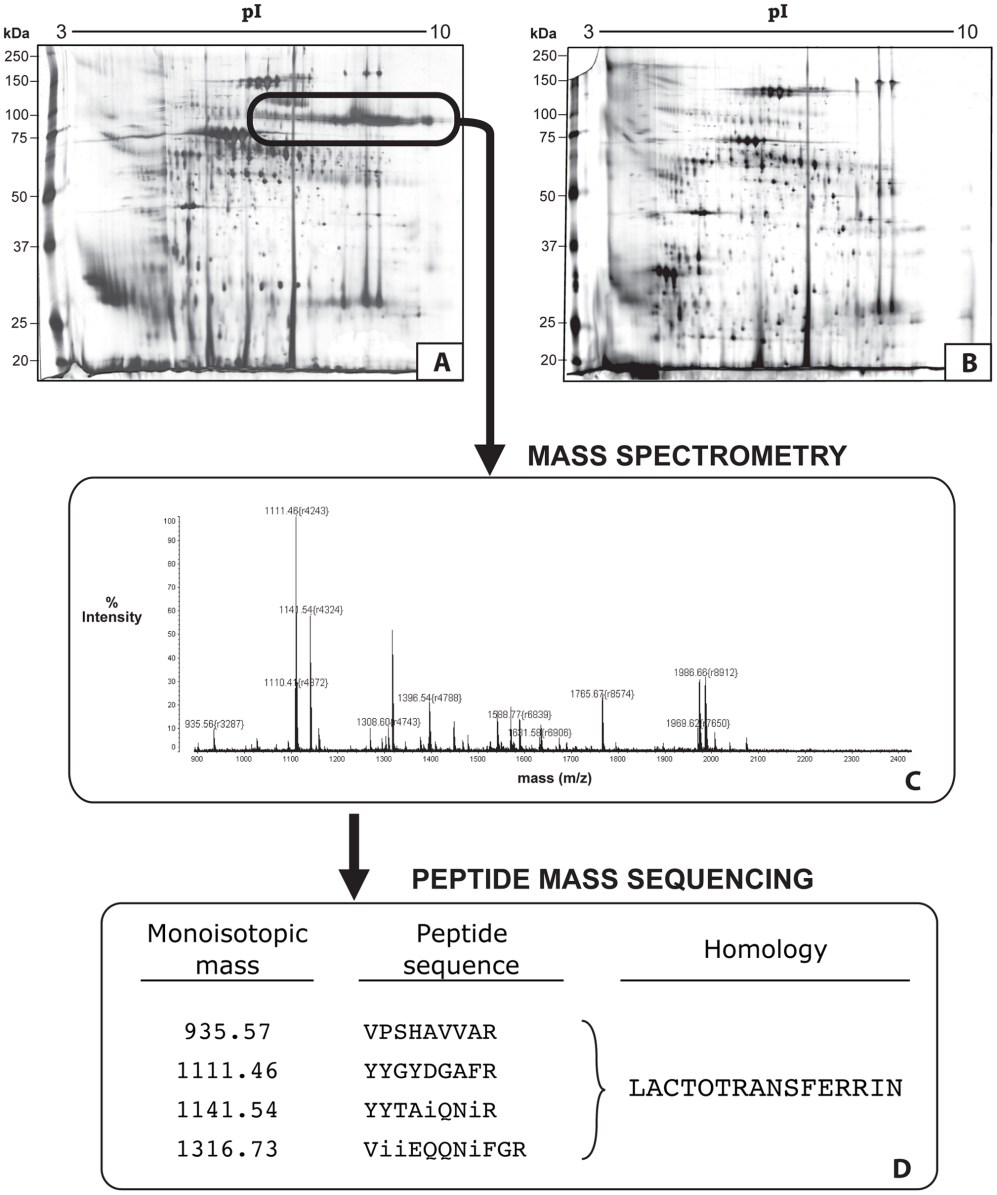
Protein separation and peptide mass sequencing of Asian elephant seminal plasma. Seminal plasma proteins were separated by two-dimensional gel electrophoresis. Selected spots were excised and submitted for mass spectrometry (MALDI-TOF) for protein identification by *de novo* sequencing of MALDI-PSD tandem mass spectra. **Panel A**: 2D gel of seminal plasma proteins from an ejaculate exhibiting good sperm motility (≥65% tMOT). The same sample from Panel A is also shown in Figure 2, Lane G5. Cored spots from within the train of this protein (circled; corresponding with band A in Figure 2) were submitted for mass spectrometric analysis. **Panel B**: Example of a 2D gel of seminal plasma proteins from an ejaculate exhibiting poor sperm motility (≤10% tMOT). **Panel C**: Mass spectrum results from MALDI-TOF. **Panel D**: Peptide mass sequencing identified the cored proteins as having homology to lactotransferrin (i  =  isoleucine or leucine).

Proteins from elephant seminal plasma (15 ‘good motility’ samples from 5 bulls; 15 ‘poor motility’ samples from 6 bulls) were also separated by 2D gel electrophoresis to allow the identification of selected proteins using *de novo* sequencing of MALDI-PSD (Post Source Decay) tandem mass spectra (Figure 3). Comparison of protein profiles between good (Figure 3A) and poor (Figure 3B) motility ejaculates revealed similar protein profiles, except for the presence of a train of ∼80 kDa proteins focusing in a series of spots between 5–10 pI (Figure 3A) in good motility samples. This corresponded with the dominant band observed in SDS-PAGE (Figure 2), and was detected in 85% of seminal plasma samples from good motility ejaculates under both electrophoresis conditions. However, 90% of samples from poor motility ejaculates lacked this protein.

#### Protein identification by mass spectrometry

**Figure 4:**
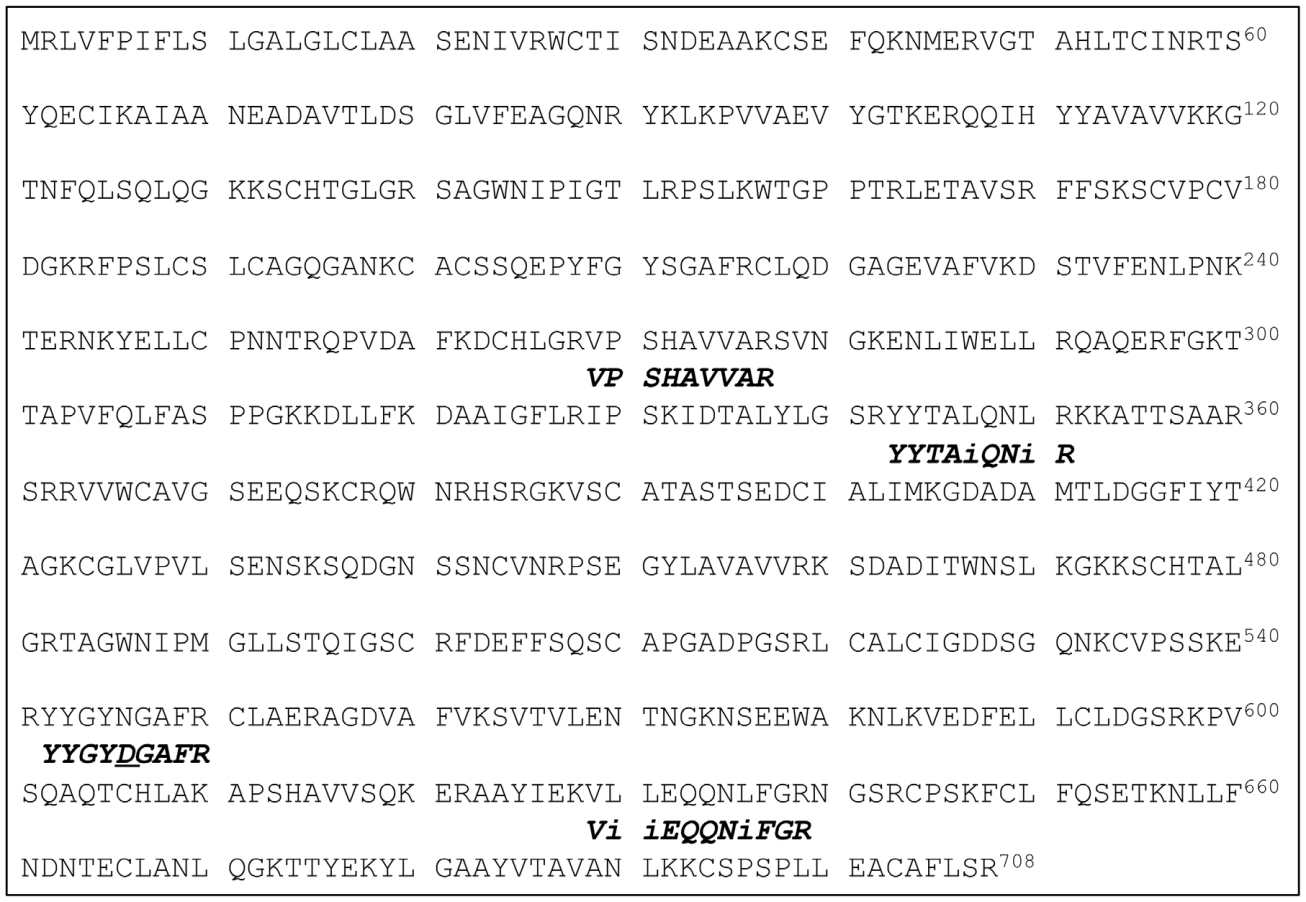
Amino acid sequence of predicted African elephant (*Loxodonta africana*) lactotransferrin determined from the 29 Mammals sequencing project (http://www.broadinstitute.org). Alignment of tryptic peptide sequences determined by MALDI-PSD sequencing of SPITC derivitized peptides shown italicized. Residues marked as “i” represent either isoleucine (I) or leucine (L), which are isobaric. Asparagine 546 has most likely been deamidated to its corresponding aspartic acid.

Trypsin digestion and mass spectrometry of the ∼80 kDa protein (Figure 3C) revealed homology to lactotransferrin (Figure 3D). Four short tryptic peptides were all found to have significant homology to lactotransferrin (Figure 3D). A BLAST search of the 4 *de novo* determined sequences against the predicted protein sequence database of African elephant (*Loxodonta africana*) found a predicted protein of 77,387 Da which matched nearly 100% of the tryptic peptide sequences (Figure 4). The only discrepancy was a single peptide which had an aspartic acid determined at a position predicted to be an asparagine. It is not uncommon for deamidation to occur on asparagine residues to convert them to the corresponding aspartic acid.

#### Immunoblotting for detection of lactotransferrin

**Figure 5:**
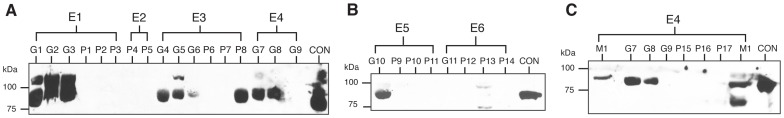
Immunodetection of lactotransferrin in elephant seminal plasma samples. Panels A, B, and C represent several immunoblots following 1D SDS-PAGE protein separation. E1-E6 denote individual Asian elephant bulls. G1 through G11 represent seminal plasma samples obtained from ejaculates exhibiting good motility (≥65% tMOT). P1 through P17 represent seminal plasma samples obtained from ejaculates exhibiting poor motility (≤10% tMOT). M1 represents a seminal plasma sample obtained from ejaculates exhibiting moderate motility (45% tMOT). CON: liver control. Approximately 85% of the seminal plasma samples from ejaculates exhibiting good motility were positive for the presence of lactotransferrin. Conversely, lactotransferrin was undetected in over 90% of seminal plasma samples from ejaculates exhibiting poor motility.

To confirm the proteomic identification, immunoblotting was used to detect lactotransferrin in seminal plasma samples from ejaculates exhibiting good versus poor motility (Figure 5). Similar to the trend observed in SDS-PAGE, the presence of lactotransferrin was confirmed in the majority of the good motility samples, while only a small percentage of poor motility samples (8%) also exhibited the presence of lactotransferrin in the seminal plasma (Figure 5).

## Discussion

A major challenge to implementing AI as a genetic management tool in elephants has been the inability to consistently collect good quality ejaculates. In this study, we analyzed the seminal plasma chemistry and protein profile of good vs. poor motility ejaculates. Results demonstrated that urine contamination alone was not always a contributing factor to poor motility. Seminal plasma pH, CPK, Na^+^, Cl^−^, and GLU, presumably from accessory gland sources, were consistently higher in good compared with poor motility ejaculates. The most surprising finding was the presence of lactotransferrin in ∼85% ejaculates with good motility compared with poor motility counterparts. These data provide the first evidence of positive correlation between presence of lactotransferrin and Asian elephant sperm motility, and suggest that the presence and/or absence of accessory gland contributions in the Asian elephant plays a profound role in explaining the variability in quality among ejaculates, even from the same bull.

With the exception of osmolality, comparison of semen characteristics between good and poor sperm motility groups exhibited significant differences among all traits. Good motility ejaculates contained higher proportions of spermatozoa that were morphologically normal and stained positive with Spermac stain. Mean ejaculate volume reported in this study was in agreement with values previously reported for the rectal massage technique [8], [9], [10] as well as other techniques including rectal massage with standing sedation [11], electroejaculation [5] and artificial vagina [7]. However, high proportion of ejaculates obtained via rectal massage represented a sperm-rich fraction with minimal seminal plasma contribution and may not represent a ‘true ejaculate’. Good motility ejaculates were characterized by larger volume and lower sperm concentration, thus indicating better quality ejaculates with higher percentages of sperm motility were diluted with larger amounts of seminal plasma. Thus, although the rectal massage technique is practical and represents a relatively safe option for semen collection from captive managed bull elephants, an alternative method that mimics natural ejaculatory responses including contributions from the accessory sex glands may be warranted to improve semen collection efficiencies in elephants.

As observed herein and in a previous study [3], a high proportion of ejaculates obtained via the rectal massage technique consistently contained spermatozoa with poor motility. However, high quality ejaculates have also been obtained utilizing the same rectal massage technique [3], [4]. This suggests that perhaps other factors, such as seasonality and/or circulating testosterone levels may contribute to this variation in semen quality [20]. Although elephants are not considered seasonal breeders, they routinely enter a cyclic heightened physiological state called ‘musth’ that is characterized by elevated testosterone levels [21]. Testosterone is necessary to maintain spermatogenesis [22] and influences semen quality in many species [15]. However, it is plausible that exponentially heightened levels of testosterone during musth may exert a detrimental effect on testicular and/or accessory gland function. In our study however, bulls were not collected during musth due to the heightened aggressive and unpredictable behavior that is associated with this physiologic state. Furthermore, daily variation in semen quality was often observed regardless of musth. We, therefore, postulate that sperm quality in elephants is influenced by other factors besides musth or changes in hormones.

Good motility ejaculates consistently expressed higher pH than their poor motility counterparts. Studies in the bull, dog, rat, hamster, guinea pig, and human species have revealed enhanced spermatozoa motility in more alkaline environments [23], and decreases in pH below physiological levels have been reported to exert a detrimental effect on sperm motility [24], [25]. The results of this study suggest that elephant spermatozoa may be highly susceptible to changes in pH resulting in a sharp decline in motility. We also found that a high proportion of ejaculates (55%) exhibited overt urine contamination, which was also observed in other studies [3], [9], [26]. Ejaculates with urine contamination results in reduced sperm motility, in large part, due to changes in pH and osmolality [24], [25], [27]. Thus, in the present study, we utilized CRT and UUN as markers for detecting low levels of urine contamination that may not have been overtly observed. We found no differences in CRT and UUN between seminal plasma from good versus poor motility samples, and there was no relationship between sperm motility (% tMOT and % pMOT) with either CRT or UUN. These results suggest that urine contamination was not the primary factor that influenced sperm motility in ejaculates utilized in the current study.

Seminal plasma enzymes, such as ALT, AST, AP, LDH, and CPK are important for sperm metabolism and regulate sperm motility, fertility, and enhanced sperm survival and thus, have been used as diagnostic markers for semen quality [15], [28]. Although we did not find significant differences in AST, AP, or LDH between good and poor motility ejaculates, our results indicated that ALT levels were significantly higher in poor motility ejaculates and were inversely associated with sperm motility and Spermac positive staining. ALT has also been used as a biomarker for cellular injury [28] and sperm membrane damage in other species including the ram [29] and rabbit [30]. It may therefore also be a useful indicator for acrosomal and/or sperm membrane integrity in Asian elephants. AP has also been utilized as an effective diagnostic marker for testicular dysfunction [31], [32], [33] due to its origin in the epididymis and testes [33], [34]. The average AP level in elephants was 500.78±615.9 (U/L) and was substantially lower compared to values reported for other species (e.g. boar [35], canine [36], stallion [33], [37], rhino [31], and beluga whale [38]). Although our results failed to find a significant relationship between AP levels and sperm quality (i.e. sperm motility, Spermac staining, normal morphology), AP, AST, and ALT, were all positively correlated to sperm concentration, suggesting these enzymes may be of testicular origin and may also serve as potential diagnostic markers for testicular function in elephants. In addition, both LDH and CPK enzymes have important roles in energy production for motility [20], [39]. We failed to find any correlation between LDH and sperm motility, but CPK was statistically higher and positively correlated with sperm motility (% tMOT and % pMOT), which suggests the enzymatic activity of CPK may influence sperm motility in elephants.

Concentrations of various ions, including Na^+^, Mg^2+^, and Ca^2+^, in seminal plasma have been suggested to be correlated with sperm motility in a number of species. Na^+^ has been implicated in regulation of sperm function, including motility [40], [41], capacitation and acrosome reaction [40]. In the present study, Na^+^ concentrations were positively correlated with sperm motility (% tMOT and % pMOT), normal morphology, and Spermac positive staining. Concentrations of Na^+^ in elephant seminal plasma was similar to values reported in stallions [37], but was much lower compared to boar, bull, dog, man, buck, and cock [15]. In addition, although Mg^2+^ plays a fundamental role in many reactions including sperm maturation, fertilizing competency, and the production of energy production for sperm motility [20], this correlation is somewhat controversial [42]. The current study found an inverse relationship between elephant seminal plasma Mg^2+^ levels and sperm motility, normal sperm morphology, and Spermac positive staining. Ca^2+^ is an important element responsible for sperm motility [43], [44] and is necessary to initiate acrosome exocytosis [45]. Although we found no statistical differences in Ca^2+^ levels between good and poor motility ejaculates, Ca^2+^ was negatively correlated with sperm motility (% tMOT and % pMOT), proportion of normal spermatozoa, and Spermac positive staining. Sivilaikul et al. [26] also observed a negative correlation between seminal plasma Ca^2+^ levels and sperm motility in Asian elephants. A recent study in mice demonstrated a similar inverse relationship between sperm motility and Ca^2+^, and determined that low calcium in seminal plasma is necessary to render sperm motile upon ejaculation [46]. Elevated levels of Ca^2+^ in poor motility ejaculates was identified to result from failure of Ca^2+^ reabsorption in the male reproductive tract [46]. Furthermore, high levels of Ca^2+^ in seminal plasma from ejaculates exhibiting poor motility also may be attributed to the leakage of intracellular Ca^2+^ from damaged or dead spermatozoa [26]. Therefore, future studies are warranted to determine whether any of these cations are themselves contributing to changes in motility or whether they reflect anomalous contributions of specific accessory sex glands.

Both glucose and fructose are the primary glycolytic sugars in seminal plasma that spermatozoa utilize as energy substrates to maintain motility [15]. Due to their crucial role in spermatozoa energy production, the measurements of these sugars have been used as diagnostic biomarkers to assess semen quality [47]. Although our study did not measure fructose in elephant seminal plasma, glucose exhibited higher values in seminal plasma from ejaculates exhibiting good motility. The average seminal plasma glucose concentration in ejaculates exhibiting good sperm motility was 6.45±9.58 mg/dl, and was substantially lower compared to man (47.17±4.13 mg/dl [48]), camel (35.8±0.9 mg/dl [49]), stallion (459±162 mg/dl [50]), and bull (128.1 – 183.1 mg/dl [51]), but was similar to the boar (1–5 mg/dl [52]), buffalo (1–20 mg/dl [53]), and ram (8 mg/dl [53]). The abundance and utilization of which type of sugar spermatozoa prefer appears to vary across species, and although glucose is the primary glycolytic sugar in stallion semen [50], [52], fructose is the primary sugar that is metabolized for energy maintenance in boar, bull, ram, and humans [52], [53], [54]. Furthermore, it has been suggested that spermatozoa prefer to metabolize glucose over fructose when spermatozoa are exposed to an equal mixture of fructose and glucose in vitro [15]. However, no information is available on selective utilization of sugars by elephant spermatozoa and this warrants further investigations.

Seminal plasma proteins have been found to influence various aspects of sperm function ([13], [55], [56], [57], [58]; among others), and specific fertility proteins have been identified in a variety of species (equine [59], bovine [60], [61], [62], porcine [63], man [64], and ovine [65]). Perhaps the most significant finding in the current study was the presence of lactotransferrin in over 85% of good motility ejaculates, which highlights its potential utility as a biomarker for ejaculate quality in Asian elephants. Lactotransferrin, also known as lactoferrin, is a glycosylated 75–82 kDa iron-binding protein that is a member of the transferrin family of proteins [66]. Lactotransferrin has been detected in various mammalian biological fluids [66], including milk, amniotic fluid, tears, and seminal plasma from several species (man [67], dog [68], boar [69], mouse [70], and stallion [68]). Although it is yet to be determined in elephants, lactotransferrin has previously been reported to be synthesized in the epididymis (mice [70], boar [69], and stallion [71]) or prostate and seminal vesicles (man [72]).

The role of lactotransferrin in biological fluids has been widely debated. It is an iron-binding protein and is involved in regulating the availability and catalytic activity of iron [66], [73]. In semen, iron serves as a catalyst in the production of reactive oxygen species (ROS) [74]. ROS in low amounts are necessary for normal sperm function [75], however, excessive amounts can be detrimental resulting in reduced sperm motility, induction of membrane lipid peroxidation, increased DNA fragmentation and ultimately premature sperm death [76]. Thus, the role of lactotransferrin in semen may be as a natural antioxidant. It has also been suggested to have antibiotic properties, conveyed by its ability to sequester iron and preventing the effects that pathogens might otherwise exert on spermatozoa [77], [78].

This study underscores the importance of determining which factors in seminal plasma influence semen quality to better enhance semen storage and preservation in this charismatic endangered species. To the best of our knowledge, this is the first detailed characterization of the chemistry and protein profiles of seminal plasma from Asian elephants and how various components correlate with semen quality. Most importantly, we found that lactotransferrin levels were positively correlated with sperm motility in Asian elephants. Further investigations are warranted to determine whether lactotransferrin itself exerts any beneficial effects on elephant sperm, and if so, to identify the molecular mechanisms involved. Additional studies to determine both the primary site of synthesis in the elephant reproductive system and whether in vitro addition of lactotransferrin would improve sperm motility in elephant ejaculates are also underway in our laboratory. Finally, our findings suggest that the current rectal massage method for elephant semen collection needs to be refined or replaced because this method produces highly variable ejaculates with significantly different seminal components between good and poor quality ejaculates.
